# Get Moving! Your Brain Will Thank You.

**DOI:** 10.1093/geroni/igab046.2885

**Published:** 2021-12-17

**Authors:** Christina Nunez, Alexandria Nuccio, Charles Golden

**Affiliations:** Nova Southeastern University, Fort Lauderdale, Florida, United States

## Abstract

Exercise and cardiovascular fitness are important for physical health and well-being. Recent studies show that exercise is associated with cognitive performance across multiple domains including memory, a common complaint for older adults. Data included a ten-word list of delayed recall, a clock drawing activity, and a three-meter walking course derived from the National Health & Aging Trends Study Database (NHATS Round 9). A total of 4977 participants were included in the analysis which was predominantly white (69.7%), non-Hispanic (94.5%), female (59.2%), and between the ages of 70-84 (62.7%). A hierarchical linear regression revealed that performance on the three-meter walking course positively predicted performance on delayed recall, F(4,3999)=300.257, p<.001, and on the clock drawing activity, which is a common screening task for cognitive decline, F(4,3978)=156.433, p<.001; accounting for 23.1% and 13.6% of the variability, respectively, over and above known demographic variables. Findings suggest that fitness may be one of many factors that is associated with memory and overall cognitive decline. These findings are timely as many individuals slowed down as a result of the COVID-19 pandemic, resulting in decreases in exercise and physical activity. Not being physically active or exercising may be related to poorer physical and cognitive health, with specific concerns regarding memory. Taking into consideration the fear and anxiety associated with declining memory in late life, it is crucial to explore this area further along with other factors that may contribute to the association and develop new ways for older adults to exercise safely during the COVID-19 pandemic.

